# Split vs. Single Bolus CT Urography: Comparison of Scan Time, Image Quality and Radiation Dose

**DOI:** 10.3390/tomography7020019

**Published:** 2021-05-20

**Authors:** Nicole Morrison, Sherrie Bryden, Andreu F. Costa

**Affiliations:** Queen Elizabeth II Health Sciences Centre, Department of Diagnostic Radiology, Dalhousie University, Halifax, NS B3H 2Y9, Canada; nc414248@dal.ca (N.M.); Sherrie.Bryden@nshealth.ca (S.B.)

**Keywords:** computed tomography urography, hematuria, radiation dose, imaging quality, time analysis

## Abstract

The purpose of this study was to compare the scan time, image quality and radiation dose of CT urograms (CTU) using a split vs. single bolus contrast media injection technique. A total of 241 consecutive CTUs performed between August 2019-February 2020 were retrospectively reviewed. There were three study groups: Group 1, <50 years old, 50/80 cc split-bolus administered at 0 and 700 s post initiation of injection, with combined nephrographic and excretory phases; group 2, ≥50 years old, same split-bolus protocol; and group 3, ≥50 years old, 130 cc single bolus injection, with nephrographic and excretory phases acquired at 100 s and 460 s post injection initiation. The recorded data elements were scan time, number of excretory phases, imaging quality based on opacification of the urinary collecting system (<50%, 50–75%, 75–100%), and dose-length product (DLP). Associations between group and categorical variables were assessed (Chi-square); mean scan time and DLP were compared (one-way ANOVA). Following analysis, proportionally fewer CTUs required a repeat excretory phase in group 3 (32/112, 28.6%) than in groups 1 (25/48, 52.1%) and 2 (37/80, 46.3%) (*p* = 0.006). Mean scan time was significantly lower in group 3 (678 s) than in groups 1 (1046 s) and 2 (978 s) (*p* < 0.0001). There was no association between groups and image quality (*p* = 0.13). DLP was higher in group 3 (1422 ± 837 mGy·cm) than in groups 1 (1041 ± 531 mGy·cm) and 2 (1137 ± 646 mGy·cm) (*p* = 0.003). In conclusion, single bolus CTU resulted in significantly fewer repeat phases and faster scan time at the expense of a slightly higher radiation dose.

## 1. Introduction

CT urography (CTU) is a valuable imaging examination for assessing a variety of conditions. The 2019 American College of Radiology (ACR) Appropriateness Criteria rate CTU as the first-line imaging examination for patients with microhematuria and risk factors for urologic malignancy, and no history to suggest a benign cause [1]. Similarly, the American Urological Association (AUA) recommends CTU in patients with asymptomatic microhematuria that persists after treatment or exclusion of any benign causes [2], and these guidelines have been endorsed by the American College of Physicians [3]. Additional indications for CTU include evaluation of patients with gross hematuria, for which the pre-test probability of malignancy is 30–40% [1], staging and surveillance of patients with urinary tract malignancy, assessing for urinary tract injury or postsurgical integrity, congenital abnormalities, and urinary obstruction [4,5].

Despite its widespread use, optimizing CTU technique is a challenge, and there is no consensus regarding a standard protocol [6]. The most common CTU technique acquires three separate phases or CT acquisitions at the following timepoints: An unenhanced phase prior to contrast media injection; a nephrographic phase acquired 80–120 s after intravenous (IV) injection of contrast media; and an excretory phase, acquired several minutes after contrast media injection, during which contrast media is excreted by the kidneys and opacifies the upper urinary tracts [6]. In this technique, the entire contrast media volume is injected as a single bolus; the advantage is that all of the contrast bolus contributes to the nephrographic and excretory phases, at the expense of three separate CT acquisitions, which results in higher ionizing radiation dose to the patient [6,7]. The second most common CTU technique is the split-bolus technique, where the nephrographic and excretory phases are acquired at the same time, in order to eliminate CT acquisition, and thereby, reduce the radiation dose by approximately one-third. Similar to the single-bolus technique, an unenhanced phase is first acquired. Then, the contrast media bolus is split into two injections. Approximately one-third of the bolus is injected several minutes prior to scanning; this portion of the bolus opacifies the urinary collecting system at the time of scanning. Approximately two-thirds of the bolus is injected 80–120 s prior to scanning; this portion of the bolus enables the kidneys to be imaged in the nephrographic phase, which corresponds to the phase when renal tumors are most evident on CT. Although the split bolus technique eliminates a third acquisition and reduces the amount of ionizing radiation, the disadvantage of splitting the bolus is that less contrast media contributes to enhancement of the kidneys and opacification of the urinary collecting system, which may degrade image quality.

Previously, our center used a split-bolus technique for CTU in all patients. However, we frequently encountered instances where the post-contrast phase did not opacify the entire upper urinary tract. These suboptimal CTUs were brought to the attention of the radiologist, who often requested a repeat acquisition to obtain better images in the excretory phase. Given that a repeat excretory phase eliminates the radiation-dose savings advantage of the split-bolus technique, we recently changed our protocol to a three-phase technique in patients ≥50 years of age. This age cut-off was chosen to balance the increased pre-test probability of upper urinary tract malignancy and need for good imaging quality in older patients [8], vs. the risk of radiation, which is higher in younger adults [9]. The objectives of this study were to compare the scan time, imaging quality, and radiation dose of the single-bolus vs. the split bolus CTU techniques.

## 2. Materials and Methods

### 2.1. Study Design

This retrospective study was approved by our institutional research ethics board (REB) as a quality assessment study. The need for full REB review and patient consent was waived. A search of the Radiology Information System was performed to retrieve all outpatient CTU examinations performed at the Queen Elizabeth II Health Sciences Centre from 20 August 2019 to 29 February 2020. This corresponded to approximately 3 months before, and after, changing our CTU protocol on 20 November 2019. Any examinations that deviated from normal protocol, such as the inclusion of additional phases or extending the z-axis range, or those degraded in imaging quality for other reasons such as patient motion or interstitial injection were excluded.

### 2.2. CT Urography Technique

CTUs were performed on three Siemens CT scanners: Definition AS+, Definition Flash and Somatom Sensation 64 (Siemens Healthineers, Erlangen, Germany). Technical parameters of the CTU technique were similar between scanners, including reference kilovoltage (100–120 kVp) and reference tube current-time product (170–190 mAs). Iterative reconstruction was available on the AS+ and Flash scanners (SAFIRE), and set to 3. For both the single and split bolus protocols, CTUs were performed with the patients in the supine position, and an initial pre-contrast phase was acquired to assess for urinary tract calculi. For the split-bolus protocol, an injection of 50 cc iohexol 350, followed by 150 cc of normal saline was injected at a rate of 3 cc per second. At 10 min after initiating the injection, a second contrast bolus of 80 cc iohexol 350 and 25 cc saline was administered. The combined nephrographic and excretory phases were imaged at 100 s thereafter, corresponding to 700 s after initiating the first contrast media injection. For the three-phase protocol, 130 cc iohexol 350 followed by 150 cc normal saline were injected at 3 cc per second. The kidneys were imaged in nephrographic phase 100 s after initiating the injection. The kidneys, ureters and urinary bladder were imaged in excretory phase 6 min, thereafter, corresponding to 460 s after initiating the first contrast media injection.

### 2.3. Data Collection

Patient and CT data were collected primarily by a fourth-year radiological technology student (NM). Image quality evaluation data was validated by a board-certified, fellowship-trained abdominal radiologist with 5 years of experience (AFC). The radiologist also applied the inclusion/exclusion criteria. For each CTU examination, the patient age, sex, date of examination, and CT scanner were recorded. The timestamps of the initial pre-contrast phase and final post-contrast phase were recorded, and the difference was taken as the total scan time. The number of post-contrast phases was recorded. For each post-contrast phase, opacification of the upper urinary tract (renal pelvicalyceal systems and ureters) was graded as follows: <50%; 50–75%; and 75–100%. The dose-length product (DLP) was recorded.

### 2.4. Statistical Analysis

Demographic information of the study cohort was calculated. The cohort was divided into the following three groups: patients <50 years old, all of whom were imaged with the split-bolus protocol; patients ≥50 years old who were imaged with the split-bolus protocol before 20 November 2019; and patients ≥50 years old who were imaged with the single-bolus protocol after 20 November 2019. Differences in scan time and DLP were assessed with one-way ANOVA, with the Tukey post hoc test for comparison between patient groups. Chi-square was used to assess for any association between patient group and sex distribution, number of excretory phases, and quality rating of excretory phases 1 and 2. Statistical analysis was performed using Prism version 8.4.1 (GraphPad Software Inc., La Jolla, CA, USA).

## 3. Results

**Figure 1:**
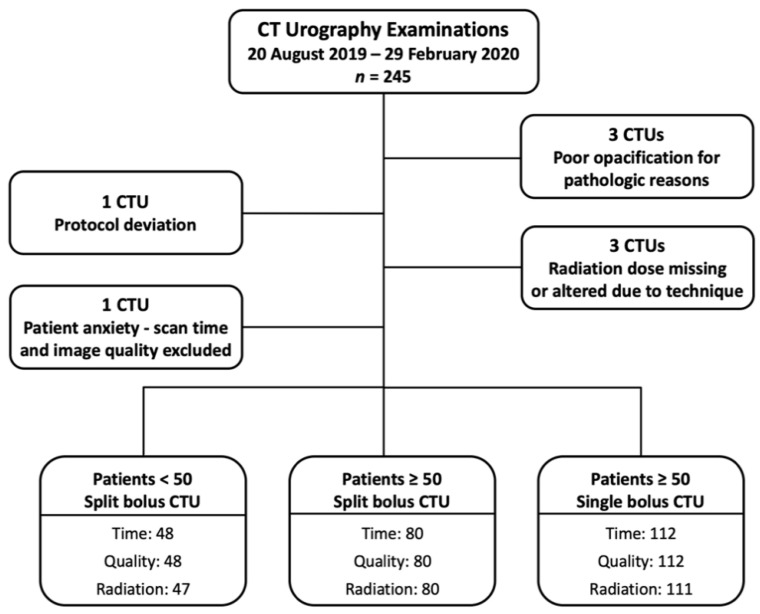
Study patient flowchart.

A flowchart of the study cohort is provided as Figure 1. There were 245 CTUs performed during the study period. Three CTUs were excluded due to the pathologic processes causing poor opacification of the urinary collecting system (two bladder tumors and one colovesicular fistula). One CTU was excluded due to protocol deviation. Three CTUs were excluded from radiation dose analysis (one dose report missing, and two imaging scan length protocol deviations). One CTU was excluded from the scan time and imaging quality assessment as the patient became anxious during examination, and the CT was temporarily halted.

**Figure 2:**
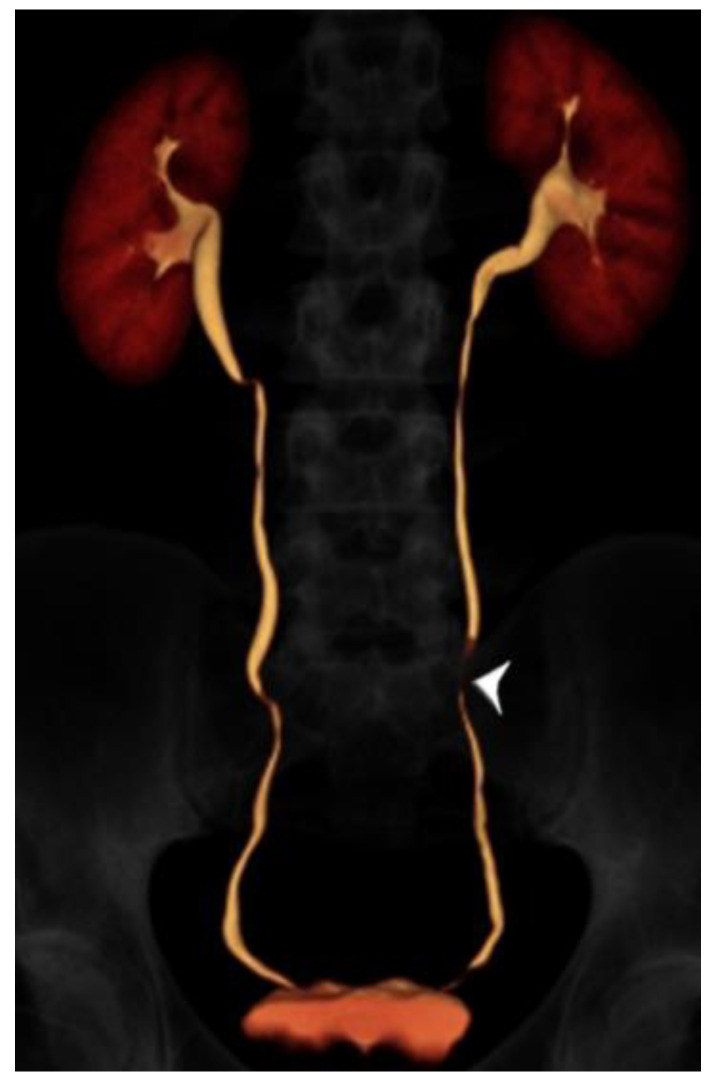
24 year-old male with gross hematuria imaged with split-bolus CT urography. Volume-rendered image of the excretory phase shows excellent opacification of the urinary collecting system. There is a very short region where the mid left ureter is not opacified by contrast (arrowhead). The total table time was 14 min and the dose-length product was 614 mGy·cm.

**Figure 3:**
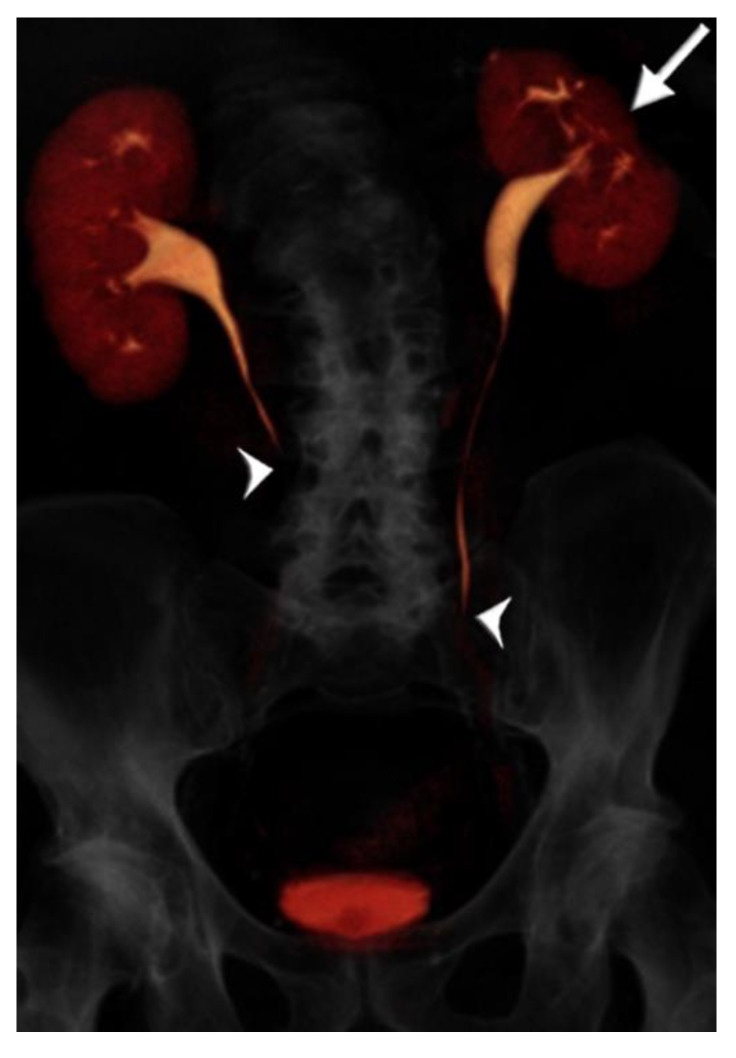
73 year-old male with microhematuria imaged with split-bolus CT urography. Volume-rendered image of the first excretory phase shows no contrast opacification of the mid-distal ureters (arrowheads). There is cortical scarring of the left kidney from previous nephrectomy for renal cell carcinoma (arrow). On repeat acquisition of the excretory phase, no improvement in image quality was achieved. The total table time was 17 min and the dose-length product was 1731 mGy·cm.

**Figure 4:**
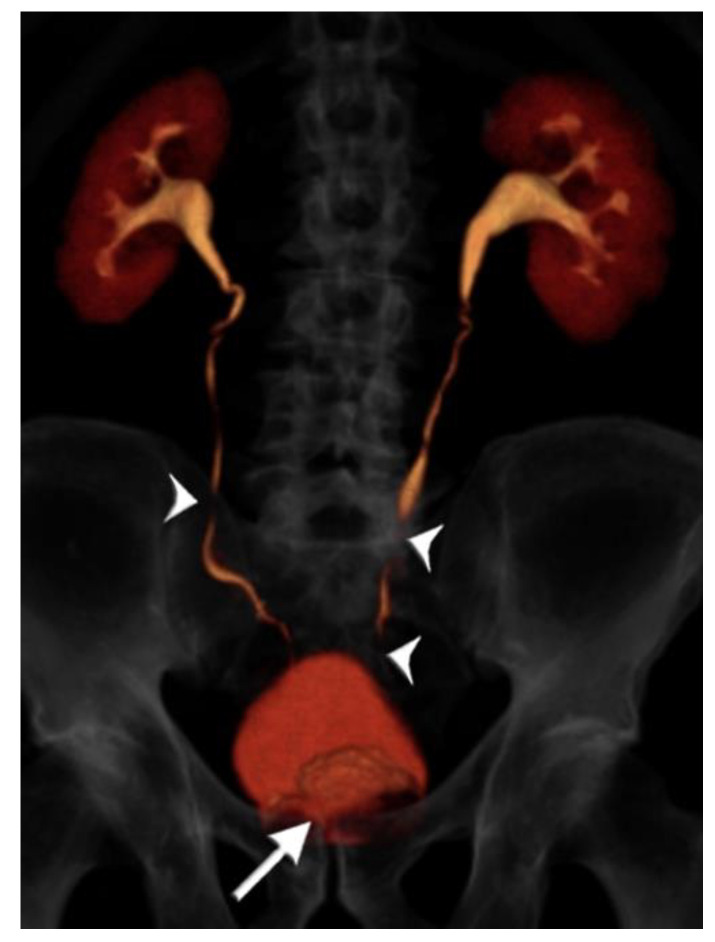
75 year-old male with gross hematuria imaged with single-bolus CT urography. Volume-rendered image of the excretory phase shows near complete contrast opacification of the ureters, apart from very short unopacified segments (arrowheads). There is an enlarged prostate indenting the urinary bladder base (arrow). There is no upstream hydroureteronephrosis and the image quality was deemed adequate. The total table time was 11 min and the dose-length product was 870 mGy·cm.

A summary of patient and CTU characteristics for each group is provided in Table 1, and examples of excretory phase volume-rendered images are provided from each group in Figure 2, Figure 3 and Figure 4. There was no association between patient group and sex distribution (*p* = 0.48). There was no statistically significant difference in mean scan times for the split bolus groups: 17 min, 26 s for the <50 year-old group, and 16 min, 18 s for the ≥50 year-old group (*p* = 0.22). The mean scan time for the single bolus group was 11 min, 18 s, and this was significantly shorter than both split bolus groups (*p* < 0.0001 for both groups).

Table 1 shows the number of CTUs in each group that required additional excretory phases. Approximately half of CTUs in both split bolus groups required at least one additional excretory phase to opacify the collecting system: 25/48 (52.1%) in the <50 year-old group and 37/80 (46.3%) in the ≥50 year-old group. In comparison, only 32/80 (28.6%) CTUs in the single bolus group required at least one additional excretory phase, and this was significantly less (*p* = 0.006). In terms of imaging quality, the degree of urinary tract opacification in most CTUs was in the 75–100% range (140/240, 58.3%) in the first excretory phase. However, 70/240 (30.0%) of CTUs were graded as 50–75% opacification, and 28/240 (11.7%) of CTUs were graded as <50% opacification. A total of 94/240 (39.2%) CTUs underwent a second excretory phase, and 7/240 (2.9%) underwent a third excretory phase. For both the first and second excretory phases, there was no association between the 3 patient groups and opacification of the urinary collecting system (*p* = 0.13, and *p* = 0.41, respectively).

The mean DLP values for the split bolus groups were not significantly different: 1041 ± 531 and 1137 ± 646 mGy·cm for the <50 and ≥50 year-old groups, respectively (*p* = 0.75). The mean DLP of the single bolus group was 1422 ± 837 mGy·cm, or 285 mGy·cm higher than the corresponding ≥50 year-old split bolus group. This was significantly higher than both the ≥50 year-old (*p* = 0.02) and <50 year-old (*p* = 0.008) split bolus groups.

## 4. Discussion

In this study, we compared the scan time, image quality and radiation dose of split vs. single bolus contrast media injection techniques for CTU. The single bolus technique was on average 5–6 min more efficient than the split bolus groups. Whereas approximately half of patients imaged with the split bolus technique required a repeat excretory phase—thereby eliminating the advantage in radiation dose savings—fewer than one-third of patients in the single bolus group required a repeat excretory phase. However, image quality was not significantly different between groups. The disadvantage of the single bolus group is a higher radiation dose: specifically, 25% more radiation (285 mGy·cm) than the ≥50 year-old split bolus group. To put this number into context, an abdominopelvic CT imparting 285 mGy·cm to a 67 year old man (the mean age in our single bolus group) corresponds to an additional effective radiation dose of 5.13 mSv and an increased cancer risk of 0.02% [10].

### 4.1. CTU Technique—Comparison to the Literature

Similar to our study, other authors have reported better contrast opacification of the urinary tract with the single bolus technique [11]. However, in another head-to-head study comparing a single-bolus three-phase technique to a split-bolus two-phase technique, Dillman et al. found that the single bolus technique yielded better distention of the urinary tract, but not better contrast opacification [12]. The split-bolus technique used by Dillman et al. was similar to the technique used in our study, with a similar delay of the combined nephrographic/excretory phase at around 12 min in both studies. However, in their protocol, slightly more intravenous saline was administered prior to the excretory phase (250 cc vs. 150 cc) and more iodinated contrast media was administered for the excretory phase (22.5 mg iodine vs. 17.5 mg). The 50–80 cc split bolus ratio used in our study has been described elsewhere [11,13], and is similar to the 3:7 ratio that Lee et al. found yielded better renal cortical enhancement over a 1:1 split bolus ratio [14]. A minority of patients in our study demonstrated poor (<50%) contrast opacification of the urinary tract: 8/48 (16.7%) in the <50 year-old split bolus group; 12/80 (15.0%) in the ≥50 year-old split bolus group; and 8/112 (7.1%) in the single-bolus group. These values are comparable to the ~15% of patients with <50% opacification in the split-bolus CTU study by Maheshwari et al. [13]. Based on the literature, it is clear that consistently opacifying the entire urinary tract at CTU remains a challenge. In addition to oral hydration and intravenous saline administration used in the CTU protocols in this study, other methods that have been proposed to improve image quality include changing patient position (such as log-rolling), abdominal compression, and the use of a diuretic [15,16,17].

### 4.2. Radiation Dose of CTU

It is important to consider the radiation dose imparted by CTU because it is routinely used to evaluate common conditions, such as asymptomatic microhematuria, but for which the prevalence of disease states (such as malignancy) which are causative is very low [1,2,18]. A recent study that modeled the risk of malignancy and associated mortality due to ionizing radiation from CTU found that, in a population of 100,000 patients with asymptomatic microscopic hematuria, there would be 53.1 and 478 patients diagnosed with upper tract urothelial carcinoma and renal cell carcinoma, respectively, but also 149 cases of radiation-induced malignancy and 101 fatalities [19]. The mean DLPs reported in our study compare favorably with what has been previously reported. For example, a recent study evaluating DLPs associated with CTU across 14 countries found a median of 1740 mGy·cm and 25th and 75th percentiles of 869 and 2943 mGy·cm, respectively [15]. We limited the radiation dose in young patients by applying the split bolus protocol to patients <50 years old, which is similar to the 40-year old cutoff applied by Cheng et al. [7]. We also limited the radiation in young patients by having the radiologist check the unenhanced phase prior to contrast media injection, as hematuria in young patients is often caused by urinary tract calculi [20]. There is growing evidence that CTU can be safely avoided in younger patients presenting with hematuria, given that the prevalence of urologic malignancy in this population is almost negligible [8,20,21]. In fact, the European Society of Urogenital Radiology guideline for hematuria avoids CTU in patients <40 years old [22]; the AUA guidelines, in contrast, recommend CTU in all patients with asymptomatic microhematuria that persists after treatment or with no attributable benign cause [2].

### 4.3. Is the Excretory Phase at CTU Necessary?

Another important consideration with respect to CTU technique is whether the excretory phases with poor urinary tract opacification should be repeated, or whether an excretory phase should be acquired at all. There are two arguments against acquiring a uniformly opacified urinary collecting system. First, the prevalence of ureteral malignancy is very low: urothelial carcinoma of the bladder is 25 times more common than that of the kidney, and 100 times more common than urothelial carcinoma of the ureter [17]. Second, when present, ureteral malignancies are evident without the excretory phase. In a study of patients that underwent CTU prior to nephroureterectomy or ureteroscopy, all resected or biopsied tumors that were located in unopacified segments of the ureter were identifiable by secondary signs, without the need for repeat excretory phases [23]. Another study of 376 patients with hematuria and negative cystoscopy found that all subsequent urothelial carcinomas (*n* = 7) and renal cell carcinomas (*n* = 4) were evident in the nephrographic phase alone; the authors suggest that CTU protocols could be simplified by excluding the excretory phase altogether [24]. In other words, optimizing ureteral opacification in order to identify subtle ureteral carcinomas that are large enough to be seen on CTU, but not large enough to distend the ureteral lumen or cause hydronephrosis, may be entirely unnecessary, given the exceedingly low probability of such lesions [17].

### 4.4. Limitations

Our study has limitations. Although similar in technique to other centers, CTU protocols vary and our protocols and patient population are not necessarily generalizable to other centers. Data regarding patient weight or body mass index were unavailable, but patient girth influences the DLP of CT examinations with automated tube current and kV settings. A potential source of bias in our study is that different radiologists were asked whether a repeat excretory phase was warranted, which may have resulted in potential variability in how radiologists request repeat phases in patients with unopacified segments of the upper urinary tract.

## 5. Conclusions

In conclusion, we found that the single bolus CTU yielded fewer repeat excretory phases, similar imaging quality and faster scan time at the expense of a 25% higher radiation dose. Radiologists at our center find it easier to report the single bolus protocol, with dedicated nephrographic phase and fewer excretory phases. Similarly, our CT technologists prefer the more efficient scan time and improved patient throughput. Irrespective of technique, opportunities remain for improvement and standardization with respect to CTU imaging quality and radiation dose savings. There are also opportunities to improve evidence-based guidelines for work-up of conditions evaluated by CTU, such as hematuria.

## Figures and Tables

**Table 1:** Summary of patient and CT Urography characteristics for each patient group.

Characteristic	A: Split Bolus Patients <50 Years Old	B: Split Bolus Patients ≥50 Years Old	C: Single Bolus Patients ≥50 Years Old	*p*-Value (One-Way ANOVA)	*p*-Value (Tukey’s Test)
Age (years), mean ± SD	38.6 ± 8.1	68.6 ± 10.1	66.8 ± 9.6	-	-
**Sex**					
Male	24	44	68	0.48	
Female	24	36	45		
Total scan time (min:s), mean ± SD	17:26 ± 44	16:18 ± 77	11:18 ± 113	<0.0001	A:B 0.22
					A:C < 0.0001
					B:C < 0.0001
**Number of excretory phases acquired**					-
1	23	43	80	0.006	
≥2	25	37	32		
**1st excretory phase contrast opacification**					-
<50%	8	12	8		
50–75%	14	28	30	0.13	
75–100%	26	40	74		
**2nd excretory phase contrast opacification**					-
<50%	2	8	8		
50–75%	9	14	13	0.41	
75–100%	14	15	11		
Total dose-length product (mGy·cm), mean ± SD	1041 ± 531	1137 ± 646	1422 ± 837	0.003	A:B 0.75
					A:C 0.008
					B:C 0.02

## Data Availability

Not applicable.
